# Localization of the Epileptogenic Zone Using High Frequency Oscillations

**DOI:** 10.3389/fneur.2019.00094

**Published:** 2019-02-12

**Authors:** Aljoscha Thomschewski, Ana-Sofía Hincapié, Birgit Frauscher

**Affiliations:** ^1^Department of Neurology, Christian Doppler Medical Center, Paracelsus Medical University, Salzburg, Austria; ^2^Department of Psychology, Paris-Lodron University of Salzburg, Salzburg, Austria; ^3^Montreal Neurological Institute and Hospital, McGill University, Montreal, QC, Canada

**Keywords:** high-frequency oscillations, epilepsy, EEG, MEG, source localization

## Abstract

For patients with drug-resistant focal epilepsy, surgery is the therapy of choice in order to achieve seizure freedom. Epilepsy surgery foremost requires the identification of the epileptogenic zone (EZ), defined as the brain area indispensable for seizure generation. The current gold standard for identification of the EZ is the seizure-onset zone (SOZ). The fact, however that surgical outcomes are unfavorable in 40–50% of well-selected patients, suggests that the SOZ is a suboptimal biomarker of the EZ, and that new biomarkers resulting in better postsurgical outcomes are needed. Research of recent years suggested that high-frequency oscillations (HFOs) are a promising biomarker of the EZ, with a potential to improve surgical success in patients with drug-resistant epilepsy without the need to record seizures. Nonetheless, in order to establish HFOs as a clinical biomarker, the following issues need to be addressed. First, evidence on HFOs as a clinically relevant biomarker stems predominantly from retrospective assessments with visual marking, leading to problems of reproducibility and reliability. Prospective assessments of the use of HFOs for surgery planning using automatic detection of HFOs are needed in order to determine their clinical value. Second, disentangling physiologic from pathologic HFOs is still an unsolved issue. Considering the appearance and the topographic location of presumed physiologic HFOs could be immanent for the interpretation of HFO findings in a clinical context. Third, recording HFOs non-invasively via scalp electroencephalography (EEG) and magnetoencephalography (MEG) is highly desirable, as it would provide us with the possibility to translate the use of HFOs to the scalp in a large number of patients. This article reviews the literature regarding these three issues. The first part of the article focuses on the clinical value of invasively recorded HFOs in localizing the EZ, the detection of HFOs, as well as their separation from physiologic HFOs. The second part of the article focuses on the current state of the literature regarding non-invasively recorded HFOs with emphasis on findings and technical considerations regarding their localization.

## 1. Introduction

Despite more than 30 antiepileptic medication available on the market (1, 2), about 30–40% of patients treated for epilepsy continue to have seizures (3). For these patients with drug-resistant focal epilepsy, surgery constitutes the most promising treatment option in order to achieve seizure freedom (4–6). The success of surgical interventions foremost depends on the localization of the epileptogenic zone (EZ), defined as the area of brain responsible for seizure generation (7). However, identifying this brain region is challenging, as all available diagnostic tools are not able to directly measure the EZ. Only *post-hoc*, after surgery, and when seizure freedom is achieved, we are able to conclude that the EZ had been within the resected area (7). Consequently, results from multiple modalities are considered in order to indirectly infer the location of the EZ. The current gold standard for identification of the EZ is the seizure-onset zone (SOZ). The fact, however that surgical outcomes are unfavorable in 40–50% of well-selected patients (8), suggests that the SOZ is a suboptimal biomarker of the EZ, and that new biomarkers resulting in better postsurgical outcomes are needed.

High frequency oscillations (HFOs) have been proposed as a promising biomarker of the EZ (9–15). HFOs are spontaneous events occurring in electroencephalography (EEG) or magnetoencephalography (MEG) signals, defined as at least four oscillations with frequencies higher than 80 Hz, which distinctively stand out from the background signal (16). HFOs are divided into three subgroups: ripples (80–250 Hz), fast ripples (250–500 Hz), and very-fast ripples with frequencies exceeding even 500 Hz (17–20). Regarding epilepsy, studies suggested that a resection of brain tissue generating high rates of HFOs may lead to good post-surgical outcome (e.g., 21–23). The possible value of HFOs recorded interictally is of special interest, as this does not require to record seizures, a process which is not only time and resource consuming, but also bearing the risk of complications due to secondary generalization after lowering the patients' antiepileptic drugs. This notion of interictal HFOs as a possible biomarker for the EZ has tipped the scale to further pursue their investigation.

To establish HFOs as clinical biomarker for epilepsy, three main issues still need to be tackled. First, evidence on HFOs as a clinically relevant biomarker stems predominantly from retrospective assessments with visual marking of HFOs, leading to problems of reproducibility and reliability (24, 25). Second, there are also physiologic, non-epileptic HFOs and their existence poses a challenge, as disentangling them from clinically relevant pathologic HFOs still is an unsolved issue with considerable influence on HFO research (26–30). Such a distinction is crucial to further investigate the clinical value of HFOs in predicting outcome after epilepsy surgery. Third, most findings on HFO research stem from invasive intracranial EEG (iEEG) obtained from patients with drug-resistant epilepsy, as part of their presurgical evaluation (e.g., 17, 31–34). Recording HFOs non-invasively via scalp EEG and MEG is highly desirable, as it would provide us with the possibility to translate the use of HFOs to the scalp in a large number of patients, and to extend its application from presurgical evaluation to monitoring of disease activity and predicting seizure occurrence in vulnerable patient populations. However, accurately recording HFOs on the scalp is problematic, regarding artifacts mimicking HFOs (35–38), and a low signal-to-noise ratio (39). Moreover, localizing the sources of HFOs obtained on the scalp is challenging.

In the first part, this review considers findings from iEEG recordings, assessing the value of HFOs for the localization of the EZ. Furthermore, technical issues regarding HFO detection, and findings regarding the appearance and location of physiologic HFOs are presented. The normative values of invasively-recorded HFOs are also discussed. In the second part, this article focuses on findings of pathologic HFOs recorded non-invasively, and discusses technical considerations regarding localization of HFOs.

## 2. High-Frequency Oscillations in the Intracranial EEG

Invasive EEG recordings performed in the context of presurgical epilepsy evaluation in people with drug-resistant epilepsy provide us with excellent data to investigate high frequencies in the EEG, as they have a high signal-to-noise ratio and are less prone to artifacts in comparison to non-invasive recording techniques. Although these results are limited to this special population, many studies point to a prognostic value of HFOs in predicting the EZ (10–15, 22, 23).

Another limitation is the difficulty to perform prospective studies within this population. A recently updated Cochrane review by Gloss et al. (40) investigated the clinical value of HFOs regarding decision making in epilepsy surgery. They identified only two prospective studies and concluded that there is not enough evidence so far to allow for any reliable conclusions regarding the clinical value of HFOs as a marker for the EZ. Despite this somewhat disillusioning result, general evidence points to a potential clinical value as outlined in detail by Frauscher et al. (16). In this section, we will (i) discuss the studies that support the identification of HFOs as biomarker of the EZ, (ii) present the few prospective trials that have been reported or that are currently being conducted, and (iii) review important aspects for the detection of HFOs. Lastly, we will (iv) review means to distinguish physiologic from pathologic HFOs and discuss normative values of HFOs.

### 2.1. HFOs as Biomarker of the EZ: Evidence From Retrospective Studies

In a meta-analysis, Höller et al. (41) investigated whether patients in whom high HFO generating areas had been resected presented a better post-surgical seizure outcome in comparison to patients in whom those areas had not been resected. They found significant effects for resected areas that either presented a high number of ripples or fast ripples. However, effect sizes were small and only eleven studies fulfilled their selection criteria (41). Since then, several studies investigated the predictive value of HFOs, showing that the resection of areas with high rates of both ictal (42–45), as well as interictal HFOs resulted in a favorable surgical outcome (46–50). Better results regarding the outcome prediction were reported for very-fast ripples than for ripples and fast ripples (19, 20), which was attributed to the possibility that very-fast ripples might be less prone to being mimicked by physiologic activity or artifacts (19). However, it was also noted that very-fast ripples have not been detected in all subjects, making it only useful for a subgroup of patients (20).

Importantly, it has been suggested, that pre-surgically assessed HFO rates might not be key in predicting seizure outcome, but that high rates recorded after resection might be an indicator of seizure reoccurrence (50–54), suggesting the importance to disconnect HFO generating networks (53). Accordingly, Weiss et al. (55) found that areas with fast ripples occurring on spikes that were not resected during epilepsy surgery were linked to a poor surgical outcome. This might also explain the high specificities occasionally reported by some studies (42, 50, 52, 56). Fedele et al. (56) for instance, reported on 20 patients with mesial temporal lobe or extratemporal lobe epilepsy who underwent resective surgery. Using a prospectively trained automated HFO detector, the authors evaluated the accuracy of HFOs in post-surgical outcome prediction, and reported a specificity of 100% in predicting surgical success by combining ripples and fast ripples as a biomarker.

Noteworthy, just this year (57) reported on results from three tertiary epilepsy referral centers, in which surgical outcome was correlated and predicted by the ratio of interictal HFO removal. They found significant correlations between the resection of high rates of ripples and fast ripples with surgical outcome. However, individual analysis suggested that HFO assessment was only associated with good surgical outcome in two thirds of their patients (57). Concordantly, also Roehri et al. (58) reported that HFOs are not better in predicting epileptogenic regions on an individual level than spikes. These discrepancies and concerns further stress the need for prospective trials.

### 2.2. HFOs as Biomarker of the EZ: Prospective Studies

Höller et al. (41), searched pubmed in February 2015 and found two prospective trials. Conducting another search using the pubmed database with the terms “High frequency oscillation,” we found 1,055 publications since 2015 (search conducted on August 22nd, 2018). Screening these articles, we could identify one additional publication on a prospective trial. Two further trials are currently being conducted, and thus are registered in trial databases (see Table 1). For this review, we considered trials only to be prospective, if they entailed that findings on HFOs were taken into consideration for surgical decision making.

**Table 1:** Prospective HFO studies.

**References**	**Patient population**	**Age range**	***N***	**iEEG method-ology**	**HFO measure-ment**	**Primary outcome**	**Secondary outcome(s)**	**Outcome**
Hirsch and Scholly (59)	Drug-resistant focal epilepsy (3 groups: FCD type I, FCD type II, non-pathologic)	>3	240	Stereotactic EEG	Interictal HFOs	Percentage of seizure-free patients 12 months after surgery	Types of seizure-onset patterns; duration of epilepsy; topographic distribution of structures (i) with high epileptogenicity, (ii) with maximal interictal HFO rates, (iii) showing interictal/preictal functional connectivity alterations	Recruiting
Leung et al. (60)	Drug-resistant epilepsy	Mean = 34	34	Subdural grids/strips	Ictal HFOs	Eligibility and favorable outcome after surgery	Comparison between visual and automated HFO analysis and to a cohort of patients without HFO analysis at all	increase in eligibility by 5–6.5% and good surgical outcome by 17–18%
Modur et al. (61)	Neocortical epilepsy and focal seizures	19–32	6	Subdural grids/strips	Visually detected ictal HFOs	Seizure outcome (at least 20 months post-surgery)	Temporal and spatial HFO characteristics; concordance with common EEG markers	Engel I: *n* = 3; II: *n* = 2; III: *n* = 1
Ramachandran Nair et al. (62)	Neocortical epilepsy and epileptic spasms	4–14	5	Subdural grids	Ictal ripples	Seizure outcome (at least 1 year post-surgery)	Concordance of HFOs with other clinical markers	seizure free: *n* = 3; 90% reduction: *n* = 1; 50–75% reduction: *n* = 1
van't Klooster (63)	Drug-resistant focal epilepsy	All ages	78	ECoG	Visually detected interictal HFOs	Post-operative outcome after 1 year, HFO- vs. spike-tailored surgery	Volume of resected tissue, neurologic deficits, surgical duration, complications, cognition, QoL	Recruiting

iEEG, intracranial EEG; HFOs, high-frequency oscillations; ECoG, intraoperative electrocorticography; QoL, quality of life

Regarding the published results, Ramachandran Nair et al. (62) reported on five children suffering from focal-onset epileptic spasms. All five children received invasive video EEG monitoring using subdural grid electrodes and resective surgery afterwards. Surgical decision was based on the findings of ictal HFOs among other criteria. After surgical resection including the HFO generating areas, all children yielded a reduction in seizure frequency or were seizure-free (see Table 1 for more details). In a second study, six children with neocortical epilepsy and unifocal seizure onsets who underwent resective surgery were investigated (61). Decisions regarding resection area and resection boundaries were based on the SOZ and on ictal HFO findings in subdural invasive recordings. Findings revealed a positive seizure outcome of Engel class I or II in five out of six children. Just recently, Leung et al. (60) reported on a cohort of epilepsy patients who received iEEG recordings, from which ictal HFOs were analyzed either visually or automatically using a wavelet-transform-based analysis approach. In comparison with a previous cohort of patients where no HFO analysis was performed, the authors reported an increase of patients eligible for resective surgery from 70 to 76.5% following wavelet-transformed HFO analysis and 75% following visual HFO analysis. Accordingly, the rate of good surgical outcome increased from 57 to 71.4% and 75%.

Regarding the ongoing studies, there are currently two large trials conducted which aim at prospectively assessing the clinical value of HFO analyses for surgical decision making. The recently started SPREAD trial is a multi-center study including several hospitals in France, where the clinical value of certain biomarkers for surgical decision making in patients with focal cortical dysplasia will be evaluated (59). The investigators plan to recruit up to 240 patients and one biomarker of interest will be the interictal HFO distribution obtained by invasive stereo-EEG recordings. The second study will assess the value of interictal HFOs for delineating the EZ in intraoperative electrocorticography (63). Surgery tailored by HFOs and surgery tailored by interictal spikes will be compared with respect to surgical outcome.

What becomes apparent upon reviewing the literature is that despite notable findings suggesting that HFOs might provide us with a valuable biomarker for epileptogenicity, there are also concerns regarding their reliability as a marker (50, 51, 53, 57). This stresses the need for prospective multi-center trials enabling clinicians to quantify a potential value. In the context of this need, it becomes important to answer the question on how we can best and least time consuming assess HFOs and how we can separate epileptic from non-epileptic physiologic HFOs.

### 2.3. Detection of HFOs

Various groups have reviewed the technical aspects of HFO detection (see e.g., 39, 64, 65). The detection of HFOs is a challenging task, mainly due to their usual low signal-to-noise ratio, their association with other epileptic activity, and the still open questions regarding their nature and definition. We can summarize the process of detecting HFOs in three steps: recording of the signals, HFO detection, and HFO validation. Here we will summarize the approaches and practical technical guidelines to execute them. When recording, we need to consider appropriate temporal and spatial sampling of the signals. For an appropriate temporal sampling, we need a recording system that allows to record at least three times the highest frequency of interest, with a low noise level for high frequencies (39, 65, 66). Regarding spatial sampling, the literature suggests that clinical SEEG electrodes are a very good option, thanks to their robust HFO measurements (65, 67–70), their safety surgical record (65, 71), and their sampling scale, which represents a good compromise between micro and macro-scales (17, 39, 65, 68, 72).

HFO detection has greatly benefitted from the development of automated detectors (see 64, for a 2016 review) (30, 54, 73–76). It is well-known that visual HFO detection is very time-consuming, and the reliability of this procedure has been questioned on several occasions (16). Just recently, Spring et al. (25) investigated the interrater reliability of visual HFO detection in iEEG recordings. Even though the experts were presented with an automatically detected set of possible HFOs, the evaluation agreement for these events was poor with a mean Cohen's Kappa of 0.4. Furthermore, it was shown that HFO rates for given recording channels vary over time, leading to inconsistent sources (77). Automatic detectors help to minimize the time required for HFO detection and to reduce the bias induced by human raters.

Many detectors work by first bandpass filtering the signal around the frequencies of interest (i.e., the ones of ripples or fast ripples). Many of them use forward and backward filtering to eliminate phase distortion (24, 78–81), and use Finite Impulse Response (FIR) filters that in comparison to Infinite Impulse Response (IIR) filters have less tendency to oscillate and have linear phase properties (64, 67). Their general aim is to differentiate the HFO events from the background activity (39). When working with existing detectors, it is important to consider that the design of automatic detectors is based on a definition of HFO, which is not yet standardized in the field. A common definition is that of events with at least four oscillations in a frequency range from 80 to 500 Hz that “distinctively” stand out from the background signal. This definition, however, lacks precision, and thus various groups applied different ways to interpret and implement it (82).

Furthermore, the detectors are optimized for the data-set for which they were designed. Therefore, to obtain good results when working with an existing detector, it is advisable to train and validate it on a data-set with similar characteristics to the one of interest (39, 81). The quality of the detections can also be affected by muscle activity, as it can contaminate the signal resulting in increased power in the HFO frequencies of interest (39, 69, 83, 84). This latter is less of a problem in iEEG. Another important aspect to consider is that the filtering process itself can produce spurious oscillations, and can therefore contaminate the data (64, 79). For example, filtering of sharp EEG events, including spikes, can result in filtering effects mimicking HFOs (39, 70, 79). To minimize the contamination introduced by the filtering process, Navarrete et al. (64) and Bénar et al. (79) give a series of recommendations to choose an adequate configuration to minimize filter distortions and to handle these artifacts, when detecting HFOs, accordingly. For clinical purposes though, Burnos et al. (85) showed that both spike-related and non-related HFOs are likewise markers of epileptogenicity.

To minimize the number of true undetected HFOs (false negatives), a typical approach is to set the automatic detector to work with high sensitivity and low specificity (24, 38, 86, 87). Given the low specificity, the next step is the validation of the automatic detections by an expert reviewer to discard false positive detections. Zijlmans et al. (65) give practical guidelines on the visual identification process for reviewers. As different reviewers might have different definitions of HFOs and training, this is a highly subjective step. A common approach to account for inter-reviewer reliability is to consider more than one reviewer, checking for consistency in the markings (24, 38, 86, 87). Nonetheless, to account for the lack of reproducibility and possible bias that comes from relying on the selection performed by an expert, there is a need for standardized automated detection strategies and the definition of a gold standard for detection (25, 39, 64, 65).

### 2.4. Physiologic vs. Pathologic HFOs

The fact that HFOs are not only pathologic in nature but also occur under physiological conditions is a further challenge when assessing the validity of HFOs as a marker for epilepsy. Distinguishing pathologic from physiologic HFOs might increase the specificity of that marker. This requires defining HFOs to be considered either being physiologic or pathologic. For instance, continuous high frequency activity in the background EEG has been suggested to reflect physiologic activity distinctive for certain brain regions, such as the hippocampus or the occipital lobe (88). Concordantly HFOs have been considered to reflect epileptic activity when observed on a flat background, and not when they are embedded in an oscillatory background (13). Just recently, Liu et al. (89) reported on a morphological difference between HFOs obtained in patients with epilepsy and healthy controls, associating stereotypical HFOs with a high degree of waveform similarity to the SOZ of patients and HFOs appearing within random waveforms to functional regions.

In addition, HFOs couple with interictal epileptiform discharges (IEDs), such as spikes, can be considered to reflect epileptic activity as there is a clear association with pathology, and indeed, they have been shown to be more specific for the SOZ than independent HFO events (90). Furthermore, it has been suggested that the physiologic nature pertains mainly to ripples and that fast ripples mostly represent epileptic activity in these areas (31, 89, 91, 92). In this context, very-fast ripples might even more exclusively reflect epilepsy-related activity, making them a very promising candidate for clinical use, when present (18–20).

There is also the possibility to identify HFOs as being physiologic by associating them with certain physiologic processes. There are, for example, certain physiologic HFOs linked to specific cognitive processes that can be observed in special conditions or can be even evoked by tasks or stimuli. The different types of physiologic HFOs are presented in Table 2. Noteworthy, in this review article we focus on oscillations with frequencies above 80 Hz only. A more detailed description of the gamma band oscillations and their role for cognitive processes are provided in a comprehensive review by Lachaux et al. (117).

**Table 2:** Different types of physiologic HFOs.

**Type**	**Localization**	**Relevant findings**
Memory-related HFOs	Hippocampus, parahippocampus, entorhinal cortex	Spontaneously and bilaterally occurring (17, 31, 68, 78); coupled with neocortical sleep spindles (93–95); occurrence rate correlates with memory performance (96–98)
Motor-related HFOs	Motor cortex, subthalamic regions	Occur over motor areas (77, 99); highly localized and movement specific (100); associated to symptoms in Parkinson's disease and tremors (101–104)
Somatosensory HFOs	Somatosensory cortex, thalamic regions	Overly the P20 and N20 components of SEPs (105–107); HFOs overlying the ascending N20 phase, possibly reflect presynaptic action potentials and are linked to arousal and critical stimuli detection (108–110); HFOs overlying the descending N20 phase may reflect bursts of inhibitory interneurons (111, 112)
Visually evoked HFOs	Occipital lobe, visual cortex	Spontaneously occurring (90, 92); possibly related to processing of visual stimuli (113–115); evokable by visual stimuli (116)

*SEPs, somatosensory evoked potentials*.

#### Distinguishing Physiologic From Pathologic HFOs

In 2013, Matsumoto et al. (118) reported on the possibility to distinguish somatosensory associated HFOs from epileptic HFOs in patients with intracranial EEG recordings. They evoked somatosensory HFOs by asking patients to press digits on a keyboard, and compared the detected events to spontaneously occurring HFOs. Pathologic HFOs were found to have lower mean frequencies but longer durations when compared to physiologically evoked events. Automated classification revealed high sensitivity and specificity in classifying pathologic HFOs. Evoked physiologic HFOs detected on electrodes within the SOZ also differed from evoked HFOs recorded from other sites and appeared to be more similar to epileptic HFOs. Comparable results have been obtained for HFOs that can be evoked by visual stimulation (116), yielding also higher frequencies and shorter durations in comparison to epileptic HFOs (118).

A recent study by Bruder et al. (119) could further show that certain features are marginally different between memory-related ripples that appear linked to sleep spindles and supposedly epileptic ripples. Spindle-linked ripples seem to be shorter and appear to have lower amplitudes (119). In addition, both spindles and physiologic HFO activity were found to be increased during the “up-state” and decreased during the “down-state” of slow oscillations during deep sleep (26, 120). Accordingly, epileptic HFOs have been shown to appear increasingly during the “down-state” or the transition to it (121). Implementing these findings into the process of detecting HFOs and classifying iEEG channels according to epileptic or non-epileptic brain regions, von Ellenrieder et al. (122) showed enhanced classification performance after considering the different coupling to slow waves.

Besides memory-related HFOs, the study of Nonoda et al. (123) indicates that HFOs recorded over somatosensory and visual cortices that seem to reflect physiologic processes are also linked to slow waves (123). In comparison to epileptic HFOs, which were found to couple with slow waves at 3Hz, these physiologic ripples were further found to couple with even slower waves at 1 Hz during sleep in a study by (116). Similar to the results presented in the study by von Ellenrieder et al. (122) also Nonoda et al. (123) found that considering the different types of slow-wave coupled HFOs and interpreting the seemingly pathologic HFO rates only, significantly increased the prediction accuracy for the SOZ.

In addition to slow wave phases, sleep stages have also been found to modulate the occurrence of HFOs. HFOs in general are considered to appear most frequently and most widespread during NREM sleep, whilst being the least frequent and most focal during REM sleep (124–127). However, there seems to be a difference between physiologic and pathologic HFOs with respect to the sleep stages. In contrast to pathologic HFOs, physiologic HFOs appear predominantly during phasic REM sleep (128) and seem to increase in rate over night during REM sleep (127). von Ellenrieder et al. (127) further found pathologic ripples and fast ripples to decrease with increased duration of sleep. Therefore, for clinical HFO evaluation, the authors suggested to analyze the night's first NREM sleep. Interestingly, it was also shown that the occurrence of pathologic HFOs in close proximity to the EZ might be less suppressed during REM sleep (126).

Although the possibility to evoke physiologic HFOs presents an exciting way to study these phenomena in more detail and to investigate possible differences to epileptic HFOs, considering the different appearance rates during certain sleep stages seems more profitable at this point. Importantly it has also been acknowledged that there are great variations and overlap in appearance and rates of physiologic HFOs with regard to the topographic location, suggesting that establishing normative values for these various appearance rates might improve the use of HFOs for clinical purposes even further (129).

#### Normative Value of HFOs

**Figure 1:**
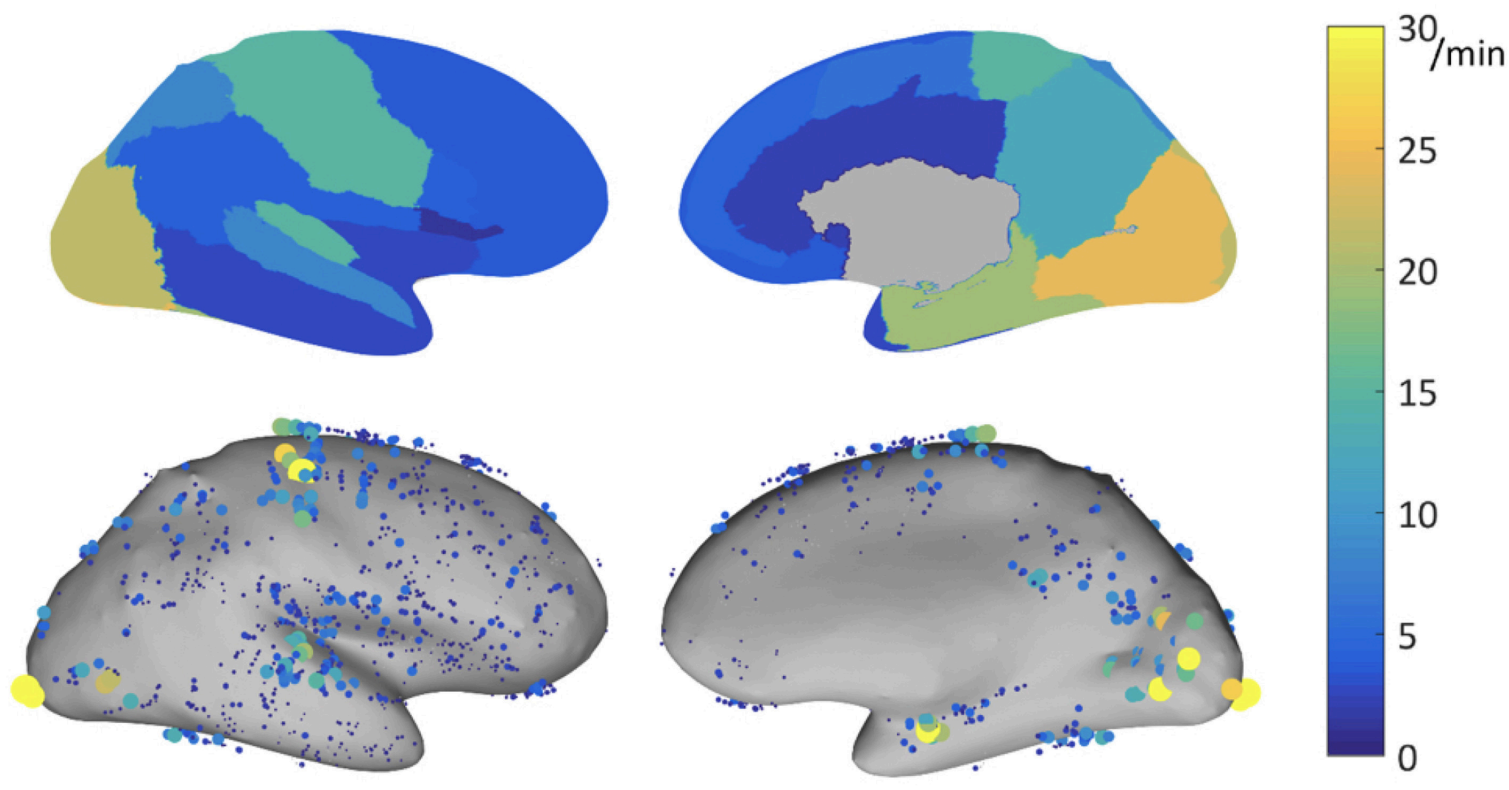
Physiologic ripple rate results for bipolar channels recorded with DIXI electrodes, represented on the inflated cortex. **Top**: 95th percentile of the physiologic ripple rate per brain region. **Bottom**: rate of the individual channels, each dot represents a channel, the size and color indicates its ripple rate (**left**: lateral view, **right**: medial view). Source: Frauscher et al. (131) with permission from Wiley.

The ability of HFOs as a biomarker for the EZ might be improved by correcting HFO rates according to their topographic localization. As mentioned before, rates of ripples vary substantially across different brain regions. A multicenter project aiming at developing normative values of iEEG activity (see 130) investigated this question by carefully selecting iEEG channels showing normal physiologic EEG activity defined as (i) absence of interictal activity during the recording period, (ii) exclusion of a significant slow wave anomaly, and (iii) being outside of lesional tissue as assessed with MRI. In a subproject of this atlas of normative iEEG activity, normative rates of HFOs (ripples and fast ripples) were assessed (131). A total of 1,171 bipolar channels with normal physiologic activity from 71 patients were analyzed. Note is made that rates of ripples varied substantially across the different regions analyzed, with rates of up to 30/min in primary eloquent cortical areas. The mean 95th percentile was 9.6/min. The highest 95th percentile rates were recorded in the occipital cortex, the medial and basal temporal region, the transverse temporal gyrus and planum temporale, the pre- and postcentral gyri, and the medial parietal lobe (see Figure 1).

The mean rate of fast ripples was very low with 0.038/min. Only 5% of channels had a rate of at least 0.2/min. This multicenter atlas is the first to provide region-specific normative values for physiologic HFOs in a common stereotactic space. It demonstrated that physiologic ripples are particularly frequent in eloquent cortical areas. In contrast, physiologic fast ripples are very rare, even in eloquent cortical areas, which makes them a better candidate for defining the EZ, when present. This atlas is an open resource available for augmentation and consultation on the web (http://mni-open-ieegatlas.research.mcgill.ca).

## 3. HFOs Obtained From Non-invasive Recordings

In the previous sections we have reviewed findings regarding invasively obtained HFOs and the possible value for presurgical evaluation in epilepsy. However, the ultimate goal for a new biomarker of epileptogenicity would be to record it non-invasively, thus sparing patients the invasive procedure of electrode implantation. Furthermore, non-invasive recordings are of interest, as they enable us to study HFOs in larger populations and not only for pre-surgical evaluation, but also for drug and disease monitoring, or even for the assessment of epileptogenic potentials after brain injury. In this section, we will present findings of ictal and interictal HFOs obtained from both EEG and MEG. Finally, we will emphasize findings regarding source localization of HFOs and review important technical considerations.

### 3.1. HFOs on the Scalp EEG

Similar to research of iEEG in the context of epilepsy, high frequencies were first investigated in the ictal state in scalp EEG recordings (132, 133). Furthermore, high frequency activity, that is frequency band power ranging above 80 Hz, rather than single HFO events, were studied first. For instance, in 2004 Kobayashi et al. (132), reported on high gamma activity of up to 100 Hz recorded during epileptic spasms in children with West-Syndrome. Comparable findings were obtained for the onset of tonic seizures in children with Lennox-Gastaut syndrome (133). Iwatani et al. (134) could show a few years later that sources of HFOs recorded at spasm onset in children with West-Syndrome spatially corresponded with cortical lesions determined by neuroimaging. A chronological list of scalp EEG studies investigating high frequency activity and later HFO events is given in Table 3.

**Table 3:** HFO investigation in epilepsy using scalp EEG.

**References**	**Patient population**	**Age range**	***N***	**Measurement**	**Detection method**	**Application/finding**
Kobayashi et al. (132)	West syndrome	3m-4y	11	Ictal HFA	Time-frequency plot	HFA during epileptic spasms and hypsarrhythmia
Kobayashi et al. (133)	Lennox-Gastaut syndrome	3y-29y	20	ictal HFA	Time-frequency plot	HFA during tonic seizure onset
Kobayashi et al. (135)	children with epilepsy and continuous spike-waves	6y-9y	10	Interictal ripples	Time-frequency plot and visual	Co-occurrence with spikes
Andrade-Valenca et al. (38)	Focal epilepsy	19y-63y	15	Interictal ripples	visual	Co-occurrence with spikes; SOZ localization accuracy for ripples 81%; lower sensitivity but higher specificity than spikes
Kobayashi et al. (136)	Childhood epilepsy with centrotemporal spikes and Panayiotopoulos syndrome	2y-9y	45	Interictal ripples	Time-frequency plot and visual	Co-occurrence with spikes; Negative correlation of HFO rates with time since last seizure
Iwatani et al. (134)	West syndrome	9m-14m	4	Ictal ripples	Time-frequency analysis and visual	HFO sources during epileptic spasms localized to lesion
Melani et al. (88)	Focal epilepsy	22y-68y	32	Interictal ripples	Visual	Ripple rates depend on spike rates; Localization of SOZ: lower sensitivity but higher specificity than spikes
Fahoum et al. (137)	Focal epilepsy	18y-43y	22	Interictal ripples	Visual	Greater thalamic BOLD changes during IEDs with high HFO rates
Lu et al. (138)	Focal epilepsy	25y-53y	5	Interictal HFA events	Visual and ICA	Association with SOZ and resection area
Zelmann et al. (139)	Epilepsy with FCD	17y-52y	11	Interictal ripples	Automated detection and visual confirmation	Proof-of-principle; SOZ identification
Chaitanya et al. (140)	Childhood/juvenile absence epilepsy	6y-10y	9	Interictal and ictal ripples	ICA, time-frequency analysis	Co-occurrence with spike-waves
Kobayashi et al. (141)	West-Syndrome	3m-9m	17	Interictal ripples	Time-frequency plot and visual	Pharmaco-response monitoring of adrenocorticotropic hormones
Toda et al. (142)	Early epileptic encephalopathy	0-17w	6	Interictal ripples	Time-frequency analysis	Co-occurrence with epileptic bursts during suppression-burst patterns
Papadelis et al. (143)	Epilepsy with encephalomalacia	11y-15y	2	Interictal ripples	Automated detection and visual confirmation	SOZ identification
Pizzo et al. (144)	Genetic generalized and focal epilepsy	21y-60y	17	Interictal ripples	Visual	Concordance between ripple-dominant hemisphere with clinical lateralization; differential diagnosis
Pizzo et al. (145)	Focal epilepsy	21y-59y	10	Interictal fast ripples	Visual	Proof-of-principle; concordance with SOZ
Qian et al. (146)	Childhood epilepsy with centrotemporal spikes	4y-11y	14	Interictal ripples	Visual	Ripple rates identified atypical forms; pharmaco-response monitoring of methylprednisolone
van Klink et al. (147)	Focal and multifocal epilepsy	18y-76y	31	Interictal ripples	Visual	Ripples preceded epileptic spikes
van Klink et al. (148)	Childhood epilepsy with centrotemporal spikes	3y-15y	22	Interictal ripples	Visual	Differentiation of atypical and self-limited forms; seizure prediction
von Ellenrieder et al. (87)	Focal epilepsy	19y-68y	17	Interictal ripples	Automated detection and visual confirmation	Localization concordance with clinical data or resected area (65% sensitivity)
Cuello-Oderiz et al. (149)	Lesional epilepsy	18y-71y	58	Interictal ripples	Visual	HFO rates higher with superficial lesions compared to deep seated focus
Mooij et al. (150)	Different types of epilepsy	11m-14y	23	Interictal ripples	Visual	Proof-of-principle; comparison with controls
Gong et al. (151)	Epileptic encephalopathy with continuous spike-and-wave during sleep	4y-13y	21	Interictal ripples	Visual	Concordance with MRI abnormalities in patients with structural etiologies; pharmaco-response monitoring under methylprednisolone
van Klink et al. (152)	Focal epilepsy	3y-42y	9	Interictal ripples	Visual	Localization concordance with clinical data or resected area (sensitivity: 55.4%, specificity: 72.2%)
van Klink (153)	Focal epilepsy	8y-54y	30	Interictal ripples	Automated detection and visual confirmation	50% localization concordance with clinical data or resected area
Bernardo et al. (154)	Children with tuberous sclerosis simplex and healthy controls	2m-5y	11	Interictal fast ripples	visual and automated detection	proof-of-principle; occurrence comparison between pediatric patients and controls
Ikemoto et al. (155)	Childhood epilepsy with centrotemporal spikes	2y-9y	25	Interictal ripples and HFA	Time-frequency analysis and Visual	Ripple rates identified atypical forms
Kobayashi et al. (156)	Myoclonic epilepsy	5m-17y	21	Ictal HFA events	Time-frequency analysis and visual confirmation	Involvement of HFA in generation of myoclonic seizures
Kuhnke et al. (157)	Epilepsies of different etiologies	8y-52y	13	Interictal ripples	Visual	Comparison of high-density and conventional EEG; higher ripple rates and better concordance with SOZ for high-density EEG
Mooij et al. (158)	Different types of epilepsy	11m-8y	23	Interictal ripples	Visual	Co-occurrence with sleep specific transients; occurrence rate during sleep stages

*m, month(s); y, year(s); w, week(s); HFA, high frequency activity; SOZ, seizure-onset zone; HFO, high frequency oscillations; BOLD, blood oxygen level-dependent; IEDs, interictal epileptiform discharges; ICA, independent component analysis; FCD, focal cortical dysplasia*.

Regarding the investigation of interictal HFOs, the first study using scalp EEG was published in 2010 by Kobayashi et al. (135). They recorded children with epilepsy and continuous spike-waves during sleep and found ripples co-occurring with epileptic spikes. In concordance with these results, the Montreal group reported for the first time an association between interictal ripples and epileptic spikes recorded non-invasively in adult patients (38). Since then several studies addressed the relationship between HFOs and IEDs (88, 137, 140, 142, 144, 147). In this context, Melani et al. (88) reported that ripple rates seem to relate to the rates of IEDs. van Klink et al. (147) further showed that ripples preceded IEDs, suggesting an interrelation between these phenomena, and excluding the possibility of these ripples to be artificially created due to filtering effects.

Importantly, when dealing with scalp EEG in the absence of iEEG findings, assessing the clinical value of HFOs with regard to the EZ becomes more difficult. In the absence of epilepsy surgery, the value of HFOs can only be assessed according to their localizing value of the SOZ or an epileptic lesion. As such, Andrade-Valenca et al. (38) investigated the localizing value of ripples for the scalp electrodes detecting the seizure onset. They found significantly more ripples on these electrodes yielding an 81% accuracy to identify the SOZ channels. Furthermore, ripples yielded a lower sensitivity but higher specificity than spikes in this context, a result that was also reported by Melani et al. (88). In order to increase the possibility to detect ripples on the scalp, using a larger coverage with more electrodes seems to be promising. Kuhnke et al. (157) just recently reported that the usage of a high-density scalp EEG with 128 electrodes did not only yield an increased detection rate of ripples, but an increased correspondence with iEEG results. Though not using source-localization in a strict sense, they were able to co-localize ripples more accurately to iEEG electrodes within areas that had been resected when using 128 electrodes as compared to only using 20 electrodes, which often led to false localizations (157).

Cuello-Oderiz et al. (149) showed that interictal scalp HFOs are predominantly recorded in epilepsy patients with superficial lesions compared to deep-seated foci. In another study, scalp HFO dominant regions were found to be concordant with MRI abnormalities in patients with structural etiologies (151). Patients with focal epilepsy were further found to have greater thalamic BOLD changes during IEDs when yielding high rates of interictal scalp HFOs accompanying those discharges (137). The occurrence of ripples was therefore associated with a more pronounced pathology of cortical-thalamo-cortical networks.

In accordance with the findings of ripples reflecting epileptogenesis, a possible application is the prediction of seizure activity. In children with Rolandic spikes, ripples were shown to predict the occurrence of seizures, and their rates differed significantly between self-limited and atypical or symptomatic courses (148). A similar observation was made by Qian et al. (146) and 2 years later by Ikemoto et al. (155), reporting on interictal ripple rates identifying atypical forms in childhood epilepsy with centrotemporal spikes. Qian et al. (146) further found interictal ripples to sensitively monitor the response to pharmacological treatment with methylprednisolone. Sensitive treatment response assessments using scalp HFOs were also reported for children suffering from epileptic encephalopathy with continuous spike-and-wave during sleep treated with methylprednisolone (151), and for children with hypsarrhythmia in West syndrome being treated with adrenocorticotropic hormones (141).

Noteworthy, there is also one report of scalp HFOs obtained in non-epileptic children by Mooij et al. (150). The authors found ripples in subjects who did not present with seizures or any interictal epileptiform activity using a standard 10–20 montage (150). This result fosters the idea of using scalp EEG not only for clinical purposes but also as a possibility to study “pure physiologic” HFOs in healthy subjects. The same authors showed that these physiological ripples were coupled to sleep-specific oscillations in children (158). On another note, all but two studies on scalp HFOs reported on frequencies below 250 Hz only. Of course, technical issues arising when trying to detect ripples are magnified for the detection of fast ripples. This is exactly the observation made by Pizzo et al. (145). They showed that a detection of frequencies >250 Hz is possible, although it is difficult and fast ripples are far less observable in scalp EEG signals than ripples due to their smaller generators and the amplifier noise at frequencies above 200 Hz (145). Just recently, Bernardo et al. (154) reported on the possibility to detect fast ripples in children with tuberous sclerosis complex. They speculate, that a detection of oscillations above the ripple band may be more feasible in children, as the skull is thinner in a pediatric population, leading to a decreased signal attenuation (154). Use of a low-noise amplifier might be helpful to overcome this challenge (56, 159).

### 3.2. HFOs in MEG

Similar to EEG, MEG recordings have an excellent temporal resolution. While EEG records electric fields that are sensitive to both tangential and radial dipole sources, MEG records magnetic fields and is sensitive to tangential dipolar sources (160) and is more selective for activity arising from fissural cortices than the EEG (161). Magnetic fields are less prone to volume conduction effects than electric fields. Therefore, MEG presents some advantages over EEG to reconstruct the neural sources responsible for the activity recorded at the scalp, which is done by means of magnetic source imaging (MSI) techniques (162).

Hand in hand with the investigation of HFOs using scalp EEG, researchers started to investigate the possibility of using MEG as well (see Table 4 for an overview). The early studies also focused on high frequency activity rather than on discrete events embedded within the MEG/EEG signals (163–165). However, Guggisberg et al. (163) showed that source localizing spike-locked beta/gamma MEG activity identified the surgically resected area in patients with a good post-surgical outcome, with an accuracy of 85%.

**Table 4:** HFO investigation in epilepsy using MEG.

**References**	**Patient population**	**Age range**	**N**	**Measurement**	**Detection method**	**Application/finding**
Guggisberg et al. (163)	Focal epilepsy	17y-67y	27	Interictal HFA	Time-frequency analysis	HFA sources identified resection area in patients with good outcome with an accuracy of 85%
Xiang et al. (164)	Lesional epilepsy	6y-17y	30	Interictal VHFA	Time-frequency analysis	Association with SOZ and epileptic lesion, proof-of-principle
Rampp et al. (165)	Focal epilepsy	20y-50y	6	Interictal HFA	Time-frequency analysis	SOZ identification in 5/6 patients (compared to invasive recording)
Xiang et al. (166)	Focal epilepsy	6y-26y	4	Ictal and interictal VHFA	Time-frequency analysis	association with SOZ and epileptic lesion, proof-of-principle
Miao et al. (167)	Childhood absence epilepsy	5y-11y	10	Ictal ripples and fast ripples	Time-frequency analysis	Identification of SOZ using source localization; Fast ripple rates corresponded with seizure frequency
Miao et al. (168)	Childhood absence epilepsy	5y-12y	14	Ictal HFO	Time-frequency analysis	Identification of SOZ using source localization
Tenney et al. (169)	Childhood absence epilepsy	6y-12y	12	Ictal HF	Time-frequency analysis	Source localization
Xiang et al. (170)	Childhood absence epilepsy	6y-10y	10	Interictal VHFA	Time-frequency analysis	Proof-of-principle; comparison with controls
Nissen et al. (171)	Focal epilepsy	6y-29y	12	Interictal ripples	Visual	Concordance between HFO and spike sources
Papadelis et al. (143)	Epilepsy with encephalomalacia	11y-15y	2	Interictal ripples	Automated detection and visual confirmation	SOZ identification
Tang et al. (172)	Childhood absence epilepsy	5y-12y	12	Ictal and interictal VHFA	Time-frequency analysis	HFO source strength correlated with seizure severity
van Klink et al. (173)	Focal epilepsy	6y-29y	12	interictal HFO	Visual	Lateralization of irritative hemisphere
von Ellenrieder et al. (87)	Focal epilepsy	19y-68y	17	Interictal ripples	Automated detection and visual confirmation	Localization concordance with clinical data or resected area (47% sensitivity)
Migliorelli et al. (74)	Focal epilepsy	6y-22y	9	Interictal ripples	Automated detection	SOZ-lobe identification with a precision of 79%
van Klink et al. (174)	Focal epilepsy	4y-29y	25	Interictal ripples	Automated detection and visual confirmation	localization concordance with clinical data; concordance with resection area in 6/8 patients, 4 achieved a good outcome
van Klink (153)	Focal epilepsy	8y-54y	30	Interictal ripples	Automated detection and visual confirmation	75% localization concordance with clinical data or resected area
Velmurugan et al. (175)	Focal epilepsy	3y-44y	20	Ictal ripples	Time-frequency analysis and visual	Localization concordance with clinical data; concordance with resection area in 6/6 patients, all achieved seizure-freedom

*m, month(s); y, year(s); HFA, high frequency activity; VHFA, very-fast high frequency activity; SOZ, seizure-onset zone; HFO, high frequency oscillations*.

When applying strict criteria for HFOs as single events, as described by Zijlmans et al. (65), interictal MEG studies reported lower event rates than in EEG recordings (87, 173). van Klink et al. (173) for example reported to find ripples only in three out of 12 patients analyzing 15 min of interictal MEG recordings. The detection rate was significantly increased when considering virtual sensors created via beamforming as compared to sensors alone (173). Especially combining methods such as beamforming with automated HFO detection algorithms resulted in a high sensitivity for interictal MEG recordings (87, 174). However, visual supervision of the automatic detection results is necessary in order to reduce false positive detections (87, 174).

Along with interictal HFO analyses, ictal MEG activity is also a subject of active investigation. Using MSI, Miao et al. (167) showed that ictal HFOs were spatially more refined than spikes and reliably localized a propagative pattern during absence seizures in childhood absence epilepsy (167, 168). Velmurugan et al. (175) just recently demonstrated the benefit of MSI in a large sample of patients with drug-resistant focal epilepsy. They were able to localize the EZ using ictal HFOs concordantly with other modalities; surgery of this identified zone performed in six patients led to seizure freedom in all of these six patients. Interestingly and differently to scalp EEG research, very high frequency components of up to 1,000 Hz have been recorded using MEG. Also, these frequencies could be localized to areas associated with the SOZ (164, 166). Xiang et al. (170) later even reported on frequencies up to 2,000 Hz. However, these studies did not investigate distinctive electrophysiological events, but merely frequency components of the recorded signals.

As revealed by studies that investigated both MEG and EEG, there are ripples observable in one modality that remain unseen in the other and vice versa (87, 153). These studies show a superior detection rate of ripples in scalp EEG. However, MEG ripple sources appeared to be more specific for the identification of epileptogenic tissue (87). Taking this into consideration, as well as the fact that source localization performance increases with the number of events, a combination of MEG and EEG might be very beneficial for both the detection and localization of interictal HFOs. Ultimately, such an endeavor is worthwhile compared to the expenditure that comes with invasive EEG monitoring and further diminishes sampling errors resulting from the restricted area investigated with intracranial electrodes.

### 3.3. Technical Issues and Obstacles of Non-invasive Recording of Scalp HFOs and Source Localization

Besides the challenges described for intracranial recording and detection of HFOs, there are other additional difficulties we need to face at the scalp level. Scalp recordings lack the excellent spatial resolution of intracranial recordings and therefore, we need to use mathematical algorithms called inverse solutions, to estimate where in the brain the signals are being generated. The whole head coverage with a high number of sensors of MEG and HD-EEG systems gives a global view of the brain activity, and a spatial sampling that is expected to facilitate the source localization procedure (compared to traditional EEG systems). Nonetheless, this high number of sensors represents a challenge for the visual detection and validation of HFOs given the amount of information needed to process. Thus, the HD-EEG and MEG HFO detection requires the implementation of automated detectors that allow to run the analysis in a suitable time frame. As in iEEG, a common approach is to use an automatic detector as a first step on the detection process, and then visually validate the detections (24, 38, 86, 87). An open question regarding the scalp spatial sampling is how many channels would be necessary to identify and localize MEG and scalp EEG HFOs.

**Figure 2:**
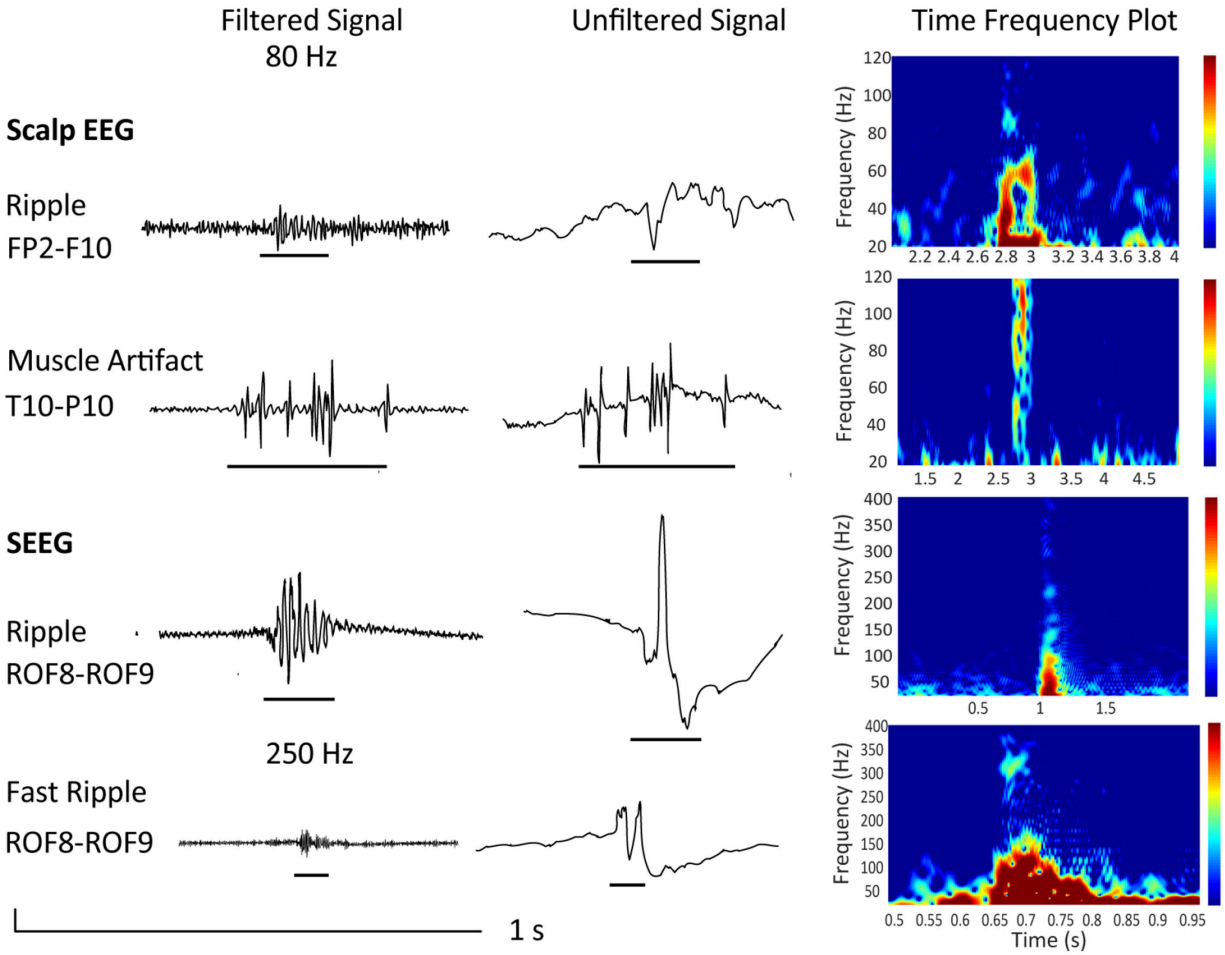
Depicted are examples from a 34 year old female patient undergoing presurgical evaluation including stereo EEG recording at the Montreal Neurological Institute and Hospital. She presented with a MRI-negative drug-resistent epilepsy and a seizure semiology suggestive of a right frontal and possible orbitofrontal generator. Scalp EEG with 25 electrodes recorded at a sampling frequency of 600 Hz showed interictal and ictal changes over right frontotemporal electrodes. Implantation showed continuous spiking over the lateral orbitofrontal region (electrode ROF 8–9). The patient underwent resection and is now seizure-free (Engel class 1) since 8 years. Neuropathology confirmed FCD IIb. Shown are a true ripple over Fp2-F10 contrasted to a muscle artifact over T10-P10 as well as a ripple and fast ripple recorded invasively at electrode ROF. All examples are given as filtered EEG signals at 80 or 250 Hz respectively, unfiltered signals, and time frequency plots. Note the isolated blobs in case of “true” HFOs.

Also, it is important to consider that artifacts produced by movement, muscle activity, and poor electrode contact have similar characteristics as HFO events (35–38). An example is given in Figure 2, which shows a “true” ripple obtained via scalp EEG and a muscle artifact, that, when filtered, mimics a ripple. Please note the difference in signal-to-noise ratio and the difference in duration in case of the muscle artifact. The figure also shows a ripple and fast ripple obtained using invasive stereo EEG for comparison. As the signal-to-noise ratio is more favorable in intracranial as compared to scalp EEG, and artifacts are more prominent in scalp as compared to iEEG, scalp EEG requires a very thorough differentiation to artifacts. As explained in section 2.4, it would be therefore advisable to assess HFOs during NREM sleep, especially when analyzing scalp EEG recordings.

Although the scalp identification of HFOs is informative, for clinical purposes we are interested in the brain areas that give rise to this activity. Reconstructing these sources constitutes an inverse problem, which requires the use of mathematical algorithms called inverse solutions to address them. Various inverse solutions exist in the literature, which mainly differ from one another by the assumptions on the neuronal sources and on the noise. Knowledge of the characteristics of the sources is therefore highly helpful when designing and implementing the inverse solution for source reconstruction (176). When source-localizing HFOs, it is important to consider that they are oscillatory transients that are not necessarily mutually phase locked, and therefore can be removed, when applying methods on averaged trials and are associated with low signal-to-noise ratio (39). Currently, there are various Open Source Software solutions for the analysis of EEG and MEG signals that include implementations of different inverse models and tutorials where the reader can have a further introduction to this subject (i.e., 177–179).

Up to now HFO source reconstruction has only been performed using MEG. The most frequently used method for HFO source reconstruction are the beamformers (74, 167, 173–175, 180, 181). The beamformers use a set of spatial filters to scan the source space. The spatial filters are designed to pass the brain activity from a specified location while attenuating activity originating at other locations. Beamformers have been widely used in the neuroscience literature to reconstruct the activity of oscillatory sources at the HFOs frequencies of interest, and they have been shown to be robust to different levels of signal-to-noise ratio (182–187). A more recent method, especially suited to localize HFO events is based on the wavelet-based Maximum Entropy on the Mean method (wMEM; 188). The wMEM was designed to localize single-trial events of oscillatory transient cortical activity which is usually associated with low signal-to-noise ratio. wMEM has been proved to correctly localize HFOs events in realistic simulations (188) and has been used to localize HFOs detected at the scalp in MEG (87, 143).

## 4. Conclusion and Future Directions

With the present article we aimed to provide a comprehensive overview of the current state of HFO research in epilepsy. There is an increasing body of evidence pointing toward the use of HFOs for delineating the EZ. However, there is still a lack of evidence derived from prospective clinical trials evaluating the clinical value of such a biomarker. Prospective trials are needed in order to assess the potential value of HFOs, especially as there are still concerns regarding the potential of HFOs as a reliable clinical marker (58). Therefore, it is indicated that conclusions of findings, especially with regard to surgical decision-making, need to be taken with caution.

Furthermore, the development and implementation of a framework for standardized HFO detection needs to be pursued, in order to reduce biases (16, 25) and make the analysis of HFOs useful in clinical routine. Therefore, automatic detectors need to be further investigated and existing algorithms need to be systematically evaluated in order to enable prolonged analysis of multiple recordings as well as the reliable detection of HFOs. The existence of physiologic HFOs in multiple areas of the brain is another obstacle that needs to be tackled. Identification of physiologic events is of special importance when it comes to source localization of HFOs as including them will obviously seriously alter the results. It awaits confirmation if normalizing HFO rates for the different brain regions as possible with the recent availability of an atlas on physiologic HFOs will indeed increase the specificity of pathologic HFOs.

Nonetheless, the increasing amount of findings suggesting also non-invasively obtained HFOs to be of use should fuel further research, as they give hope that localized sources of pathologic HFOs might improve guidance for resective surgery in the future and spare iEEG recordings. Novel markers such as very-fast ripples of up to 2,000 Hz (18–20), and more advanced analyses considering the network properties of HFOs (189–191) provide further exciting novel approaches for future research.

## Author Contributions

AT wrote the initial draft of the manuscript. A-SH provided the sections concerning technical issues and source localization. BF provided both figures, and the input with regard to the general content and structure of the manuscript. All authors together planned the manuscript, critically revised the initial draft and made final improvements prior to submission.

### Conflict of Interest Statement

The authors declare that the research was conducted in the absence of any commercial or financial relationships that could be construed as a potential conflict of interest.
